# Design, Synthesis, Molecular Dynamics Analysis, and Biological Evaluation of New Urea Derivatives in an Animal Model of Alzheimer Disease

**DOI:** 10.5812/ijpr-172672

**Published:** 2026-08-16

**Authors:** Sara Heidarian, Mehrdad Faizi, Mehrdad Alemi, Amir Garmabdari, Noushin Nikray, Mona Khoramjouy, Zahra Hajimahdi, Afshin Zarghi

**Affiliations:** 1 Department of Pharmaceutical Chemistry, School of Pharmacy, Shahid Beheshti University of Medical Sciences, Tehran, Iran; 2 Department of Pharmacology and Toxicology, School of Pharmacy, Shahid Beheshti University of Medical Sciences, Tehran, Iran; 3 Phytochemistry Research Center, Shahid Beheshti University of Medical Sciences, Tehran, Iran; 4 Department of Medicinal Chemistry, School of Pharmacy, Shahid Beheshti University of Medical Sciences, Tehran, Iran

**Keywords:** Urea Derivatives, GSK-3β, Docking Studies, Molecular Dynamics, Alzheimer’s Disease

## Abstract

**Background:**

Alzheimer disease (AD), first described by Alois Alzheimer more than a century ago, remains a major global health challenge. Glycogen synthase kinase-3β (GSK-3β) plays an important role in AD pathophysiology. Dysregulation of GSK-3β is associated with beta-amyloid accumulation, tau hyperphosphorylation, neurodegeneration, and cognitive impairment.

**Objectives:**

This study aimed to design and synthesize 17 arylalkyl urea derivatives as GSK-3β inhibitors and evaluate them in an animal model of AD.

**Methods:**

New arylalkyl derivatives were designed on the basis of the pharmacophores of GSK-3β inhibitors. Molecular docking and molecular dynamics simulations were used to evaluate the interactions of the designed compounds within the ATP-binding pocket of GSK-3β. The compounds were then synthesized by reacting substituted anilines with benzyl isocyanate, and their structures were confirmed by infrared spectroscopy, nuclear magnetic resonance spectroscopy, and liquid chromatography–mass spectrometry. The radial arm water maze was used to assess the effects of a selected compound on learning and memory deficits in an intracerebroventricular streptozotocin-induced rat model of sporadic Alzheimer-like disease.

**Results:**

Structure–activity relationship analysis indicated that variations in the R_1_ and R_2_ substituents played a crucial role in modulating GSK-3β inhibitory activity. Docking studies demonstrated that all novel derivatives fit well within the active site, with Val135 and Lys85 contributing prominently to ligand stabilization. On the basis of the docking results, compound 3b was selected for behavioural testing. Compound 3b significantly ameliorated spatial learning and memory deficits in a rat model of AD.

**Conclusions:**

Compound 3b emerged as a promising lead in this series, combining favorable GSK-3β binding properties with robust cognition-enhancing effects in vivo. These findings may inform therapeutic design, clinical translation, and strategic development in the evolving field of AD drug discovery.

## 1. Background

Alzheimer disease (AD) was first described by Alois Alzheimer in 1906 (1). It is the primary cause of dementia, accounting for approximately 60%-70% of cases worldwide (2). More than 55 million people are currently affected, and this number is expected to increase to 139 million by 2050. Despite advances in understanding its complex biology, currently approved treatments mainly control symptoms (3). In 2025, the AD drug-development pipeline included 138 investigational agents across 182 clinical trials; however, only a few have received approval from the US Food and Drug Administration, highlighting the urgent need for effective disease-modifying therapies (4). Numerous hypotheses have been proposed to explain the underlying processes, but none provides a complete explanation. Current research addresses neuroinflammation, oxidative stress, lipid and metabolic dysregulation, neurovascular unit dysfunction, and protein misfolding, seeding, and spread (5). Particular attention has been given to amyloid beta (Aβ) deposition and hyperphosphorylated tau (2, 6, 7).

AD is characterized by 2 major pathological features in the central nervous system: extracellular Aβ plaques and intracellular neurofibrillary tangles (NFTs) composed of hyperphosphorylated tau proteins (8, 9). Current pharmacological approaches to AD target Aβ plaques and NFTs (10).

Kinases involved in these pathological processes include GSK-3β and cyclin-dependent kinases. Phosphatases, such as protein phosphatase 2A, are also important, and reduced protein phosphatase 2A activity can exacerbate tau hyperphosphorylation (11, 12). Tau phosphorylation is increasingly recognized as a key factor in NFT formation, synaptic dysfunction, and neurodegeneration (13). GSK-3β plays an important role in both major pathologies of AD (14, 15). In tau pathology, GSK-3β induces aberrant tau hyperphosphorylation, thereby facilitating the formation of paired helical filaments and NFTs (16). Several studies have emphasized the direct association between NFT development and GSK-3β, one of the principal kinases involved in tau hyperphosphorylation (17, 18).

GSK-3β was discovered in 1970 (19). It is a serine/threonine kinase that plays a crucial role in neurological diseases and is therefore a promising drug target (20). Two GSK-3 isoforms have been identified: GSK-3α, with a molecular weight of 51 kDa, and GSK-3β, with a molecular weight of 47 kDa (21, 22). These isoforms share 84% overall identity and more than 98% identity within their catalytic domains (23). Their primary structural difference is the glycine-rich loop in the N-terminal region of GSK-3α (24, 25). Various small molecules have recently been identified as inhibitors of GSK-3β activity.

GSK-3β inhibitors can be classified as ATP-competitive, allosteric, non-ATP-competitive, or proteolysis-targeting inhibitors (13, 20).

Although various GSK-3β inhibitors have been designed and synthesized, only a few are currently undergoing clinical evaluation (26). This may be attributable to poor selectivity because the human kinome contains more than 500 protein kinases with highly conserved catalytic ATP-binding pockets (27). Kinase selectivity is therefore a major challenge in protein kinase inhibitor design (28).

Accordingly, we designed a novel library of arylalkyl urea derivatives as GSK-3β inhibitors. The design strategy involved hybridization of tideglusib (29) and AR-A014418 (5), 2 established GSK-3β inhibitors whose clinical development has been limited by an unfavorable balance between potency and selectivity (30). AR-A014418 served as the principal structural template because its cocrystal structure with GSK-3β shows that the 2-nitro-5-thiazolyl moiety forms key hydrogen bonds with Val135 and Asp200, whereas the urea linker interacts with the Pro136 and Val135 backbone (23). These interactions identify the urea-based scaffold as a suitable core for preserving hinge-region recognition while permitting further structural optimization (31).

**Figure 1.**
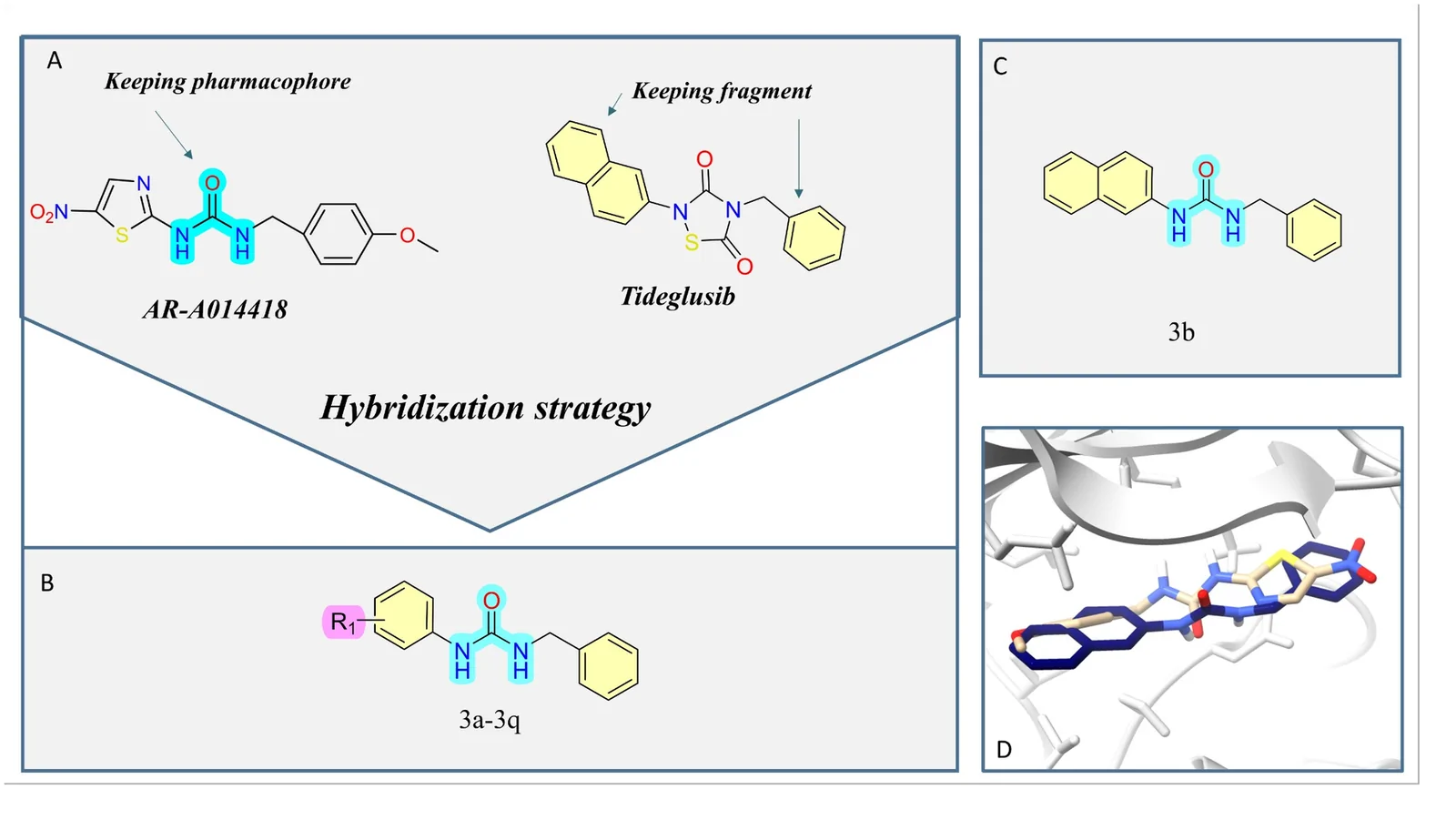
A, B, Rational design strategy for novel arylalkyl urea derivatives. Modification of the AR-A014418 scaffold to optimize hydrophobic-pocket occupancy, enhance selectivity over cyclin-dependent kinase 5 (CDK5), and improve central nervous system penetration; C, Lead compound 3b; D, Superimposition of AR-A014418 and compound 3b in the active site.

To improve blood-brain barrier (BBB) permeability, the polar nitro-containing region of the reference scaffold was replaced with a naphthyl group (R_1_) to reduce polarity and topological polar surface area while enhancing hydrophobic complementarity within the binding pocket. In parallel, the methoxy-substituted aromatic region of AR-A014418, which is solvent-exposed and contributes minimally to direct target binding, was selected as a second modification site. This region was diversified through R_2_ substitution to explore the structure-activity relationship and optimize target engagement (Figure 1).

## 2. Objectives

This study aimed to design and synthesize a series of 17 arylalkyl urea derivatives based on the pharmacophores of GSK-3β inhibitors. Docking analysis and molecular dynamics simulations were conducted to examine the interactions between the designed compounds and the GSK-3β active site. The compound with the most favorable docking score was selected for in vivo evaluation. The radial arm water maze was used to assess its effects on learning and memory deficits in an intracerebroventricular streptozotocin-induced rat model of sporadic Alzheimer-like disease. The biological activities of the synthesized compounds were evaluated using this model.

## 3. Methods

### 3.1. Materials and Instruments

All chemicals and solvents were obtained from Sigma-Aldrich and Merck AG and were of synthesis grade. When required, solvents were dried using standard procedures. Fourier transform infrared spectra were acquired using a Cary 630 FTIR spectrometer. ^1^H nuclear magnetic resonance (NMR) spectra were recorded on a Bruker FT-400 MHz instrument (Bruker Biosciences, USA). Deuterated chloroform (CDCl_3_) and dimethyl sulfoxide-d_6_ (DMSO-d_6_) were used as solvents. Splitting patterns were designated as singlet (s), doublet (d), triplet (t), quartet (q), broad singlet (bs), and multiplet (m). Liquid chromatography-mass spectrometry was performed using a 6410 Agilent triple-quadrupole mass spectrometer equipped with an electrospray-ionization interface. Elemental analysis was conducted using a Costech elemental analyzer (Italy). Reactions were monitored by thin-layer chromatography on 3 × 6-cm, 0.25-mm silica gel 60-F plates.

### 3.2. General Procedure for the Synthesis of Compounds 3a-Q

Compounds 3a-q were prepared using a one-pot procedure with aniline reagents 1a-q (0.09 mL, 0.90 mmol) and benzyl isocyanate (0.12 mL, 0.70 mmol) in a CHCl_3_/THF mixture (10 mL) under ice-bath cooling. The reaction mixture was stirred for 15 minutes to 3 hours, and reaction progress was monitored by thin-layer chromatography. After completion, the precipitated solids were removed by filtration. The filtrate was then washed with diethyl ether and n-hexane.

Detailed ^1^H and ^13^C NMR spectral data for all synthesized compounds are provided in the Supplementary Information.

### 3.3. Molecular Docking Study

Molecular docking simulations of 17 derivatives as GSK-3β inhibitors were conducted using UCSF Chimera version 1.19. The protein structure was retrieved from the Protein Data Bank (PDB ID: 1Q5K, chain A) and prepared by removing all water molecules except those essential for protein-ligand interactions, as well as the cocrystallized ligand. Atomic charges were assigned using the Kollman method, and the file was saved in PDBQT format. A 20 × 20 × 20 Å^3^ grid box, centered on the ATP-binding pocket at X = 23.99, Y = 22.82, and Z = 8.88, was used (32). Three-dimensional ligand structures were generated in MarvinSketch version 5.7 and saved in PDB format, and Gasteiger charges were assigned. Conformational searches used the default exhaustiveness setting of the Vina algorithm. Parameter files for receptor and ligand docking were prepared and integrated. Docking results were ranked by binding energy to identify the most favorable conformations and ligand orientations (33). Final docking interactions were visualized using Discovery Studio Visualizer version 4.5 and UCSF ChimeraX version 1.10.

### 3.4. Molecular Dynamics Simulation

All molecular dynamics simulations were performed using GROMACS version 2020.4. The protein-ligand complex was embedded in a cubic water box using the SPC water model, maintaining a minimum distance of 1.2 nm from the box edges. The system charge was neutralized by adding appropriate ions (Na⁺ or Cl⁻). OLSA-AA force-field parameters were used for all atoms. Energy minimization was performed using the steepest-descent algorithm (34) until the maximum force was below 1000 kJ/mol/nm. The system then underwent two-phase equilibration: an NVT ensemble at 300 K for 500 ps using a V-rescale thermostat, followed by an NPT ensemble at 1 bar for 500 ps using Parrinello-Rahman pressure coupling. The production simulation was extended to 100 ns with a 2-fs integration time step using the leap-frog algorithm (35). Periodic boundary conditions were applied in all directions, and bond constraints were handled using the LINCS algorithm. Trajectory frames were recorded at 10-ps intervals. Binding free energy was calculated using the Molecular Mechanics/Generalized Born Surface Area (MM-GBSA) method. Root mean square deviation (RMSD) and root mean square fluctuation (RMSF) analyses were performed to assess system stability.

### 3.5. In Vivo Evaluation

Male Sprague-Dawley rats weighing 200 - 250 g were housed under controlled laboratory conditions (22 ± 2 °C, a 12-hour light/12-hour dark cycle, and free access to food and water). All experimental procedures were conducted in accordance with institutional guidelines for the care and use of laboratory animals.

Animal studies were conducted in accordance with the standards of the Iranian Ministry of Health and Medical Education for the treatment and use of laboratory animals. The study was approved by the Ethics Committee of Shahid Beheshti University of Medical Sciences under approval code IR.SBMU.PHARMACY.REC.1403.326.

#### 3.5.1. Behavioral Assessments

Thirty-two male Sprague-Dawley rats weighing 200 - 250 g were obtained from the Pasteur Institute, Tehran, Iran. An AD model was induced by bilateral intracerebroventricular injection of streptozotocin (STZ; 3 mg/kg) (36, 37). The animals were divided into 4 groups: group I, control (no surgery); group II, sham (intracerebroventricular normal saline and oral gavage of vehicle for 20 days); group III, STZ (a single intracerebroventricular dose of STZ at 3 mg/kg and oral gavage of vehicle for 20 days); and group IV, STZ + compound 3b (a single intracerebroventricular dose of STZ at 3 mg/kg and compound 3b at 10 mg/kg/d for 20 days).

##### 3.5.1.1. Radial Arm Water Maze

Spatial learning and memory were evaluated using the radial arm water maze (RAWM) over 3 consecutive days, including 2 training days and 1 probe-test day. The apparatus consisted of a 6-arm water tank maintained at 25 °C, with visual cues positioned at the end of all 6 arms. Each rat was assigned a random starting arm and a target platform and was allowed approximately 60 seconds to locate the target arm. Escape latency and reference-memory errors were recorded in each trial. During the probe trial, the latency to reach the target arm, the number of entries into the target arm, and the time spent in the target arm were measured (38).

##### 3.5.1.2. Open-Field Test

Locomotor activity was assessed in a square open-field apparatus. Each rat was placed in the center and allowed to explore freely for 10 minutes. Total distance traveled and velocity were recorded. The arena was cleaned with 70% ethanol between trials to remove olfactory cues.

##### 3.5.1.3. Statistical Analysis

Data were analyzed using one-way analysis of variance and two-way analysis of variance followed by Tukey and Bonferroni post hoc tests, respectively, in GraphPad Prism version 8. Results are presented as mean ± SEM, and P < 0.05 was considered statistically significant.

## 4. Results

### 4.1. Chemistry

**Figure 2.**
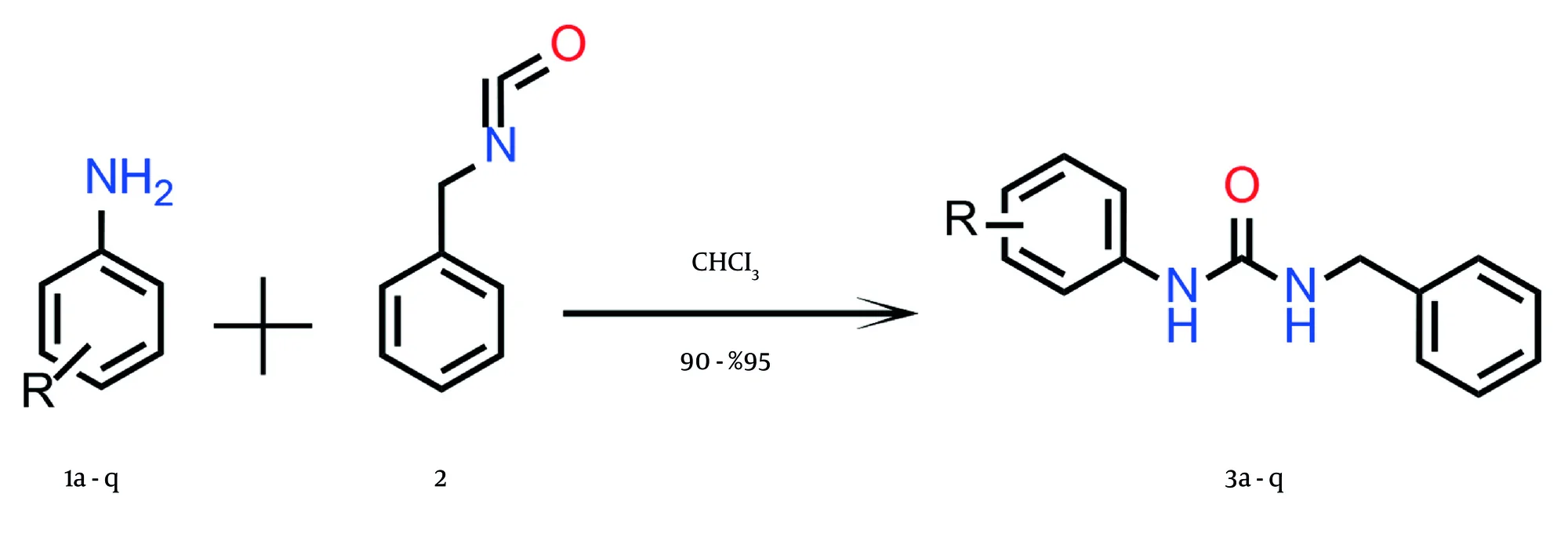
General synthetic route for preparing 1-benzyl-3-phenylurea derivatives (3a-q) through the reaction of substituted anilines (1a-q) with benzyl isocyanate (2) in CHCl_3_, yielding the corresponding urea products (3a-q) in 90% - 95% yield.

A series of 1-benzyl-3-arylurea derivatives (3a-q) was synthesized according to the route shown in Figure 2. The target compounds were obtained by reacting various aryl amines (1a-q) with benzyl isocyanate in a CHCl_3_/THF mixture under ice-bath cooling. All final products were purified and characterized by infrared spectroscopy, ^1^H NMR, ^13^C NMR, liquid chromatography-mass spectrometry, and elemental analysis.

The successful formation of the urea scaffold was confirmed by key signals in the ^1^H NMR spectra. Specifically, in the representative compound, the methylene protons (CH_2_) appeared as a doublet at 4.30 ppm, showing vicinal coupling with the neighboring amide proton. This amide proton (NH-CH_2_) appeared as a triplet at 6.81 ppm. The more deshielded NH proton attached to the aromatic ring appeared as a characteristic singlet at 8.93 ppm. These findings were consistent with the expected urea framework and confirmed the identity of the target molecules (Figure 2).

### 4.2. Molecular Docking Study

The design strategy aimed to generate arylalkyl urea derivatives with an improved balance of binding potency, kinase selectivity, and BBB permeability (12).

To validate the docking protocol, the native cocrystallized ligand TMU [N-(4-methoxybenzyl)-N'-(5-nitro-1,3-thiazol-2-yl)urea] was extracted and redocked into the active site. The resulting docking pose was compared with the original crystal structure and yielded an RMSD of 1.80 Å, confirming the validity of the docking method.

The key hinge-binding interactions of AR-A014418 were preserved, while selectivity and central nervous system-relevant physicochemical properties were improved through R_1_ and R_2_ substitution. Retention of the urea pharmacophore was expected to maintain the critical donor–acceptor interactions required for GSK-3β recognition, whereas hydrophobic substitutions at R_1_ and R_2_ were designed to improve pocket occupancy and reduce excessive polarity. These modifications were also intended to enhance selectivity over related kinases, such as CDK5, by improving hydrophobic complementarity within the GSK-3β binding site.

**Figure 3.**
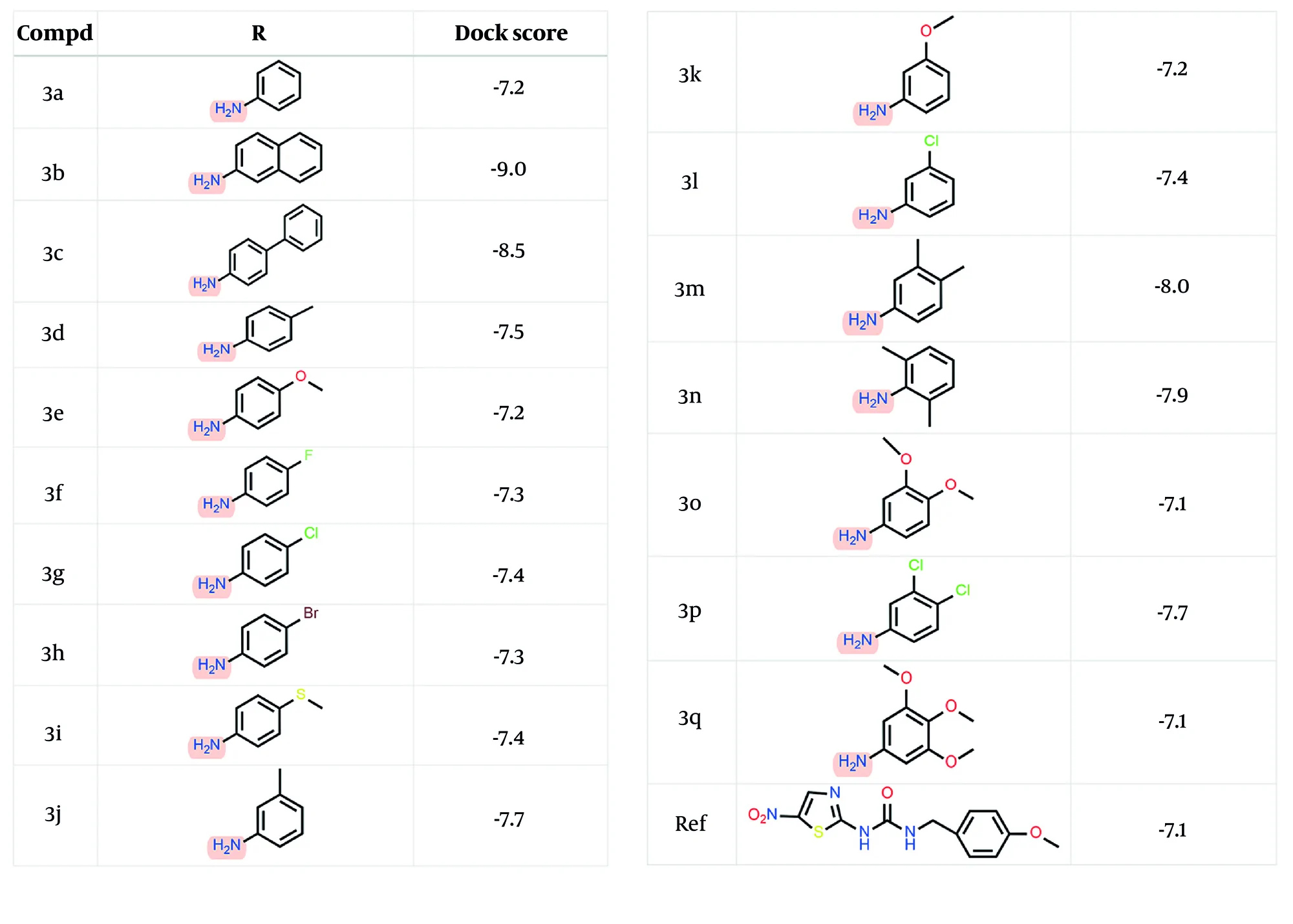
Docking scores for compounds 3a-q and the reference ligand (kcal/mol).

Molecular docking showed that all designed compounds were appropriately positioned in the ATP-binding pocket of GSK-3β and exhibited binding orientations characteristic of ATP-competitive type I inhibitors. Docking scores ranged from -7.1 to -9.0 kcal/mol (Figure 3). Compound 3b had the most favorable score (-9.0 kcal/mol), followed by compounds 3c (-8.5 kcal/mol) and 3m (-8.0 kcal/mol). The reference compound had a docking score of -7.1 kcal/mol. These results were consistent with the interaction analysis, in which bulkier or better-oriented aromatic systems, including 3b, 3c, and 3m, showed more favorable docking scores.

**Figure 4.**
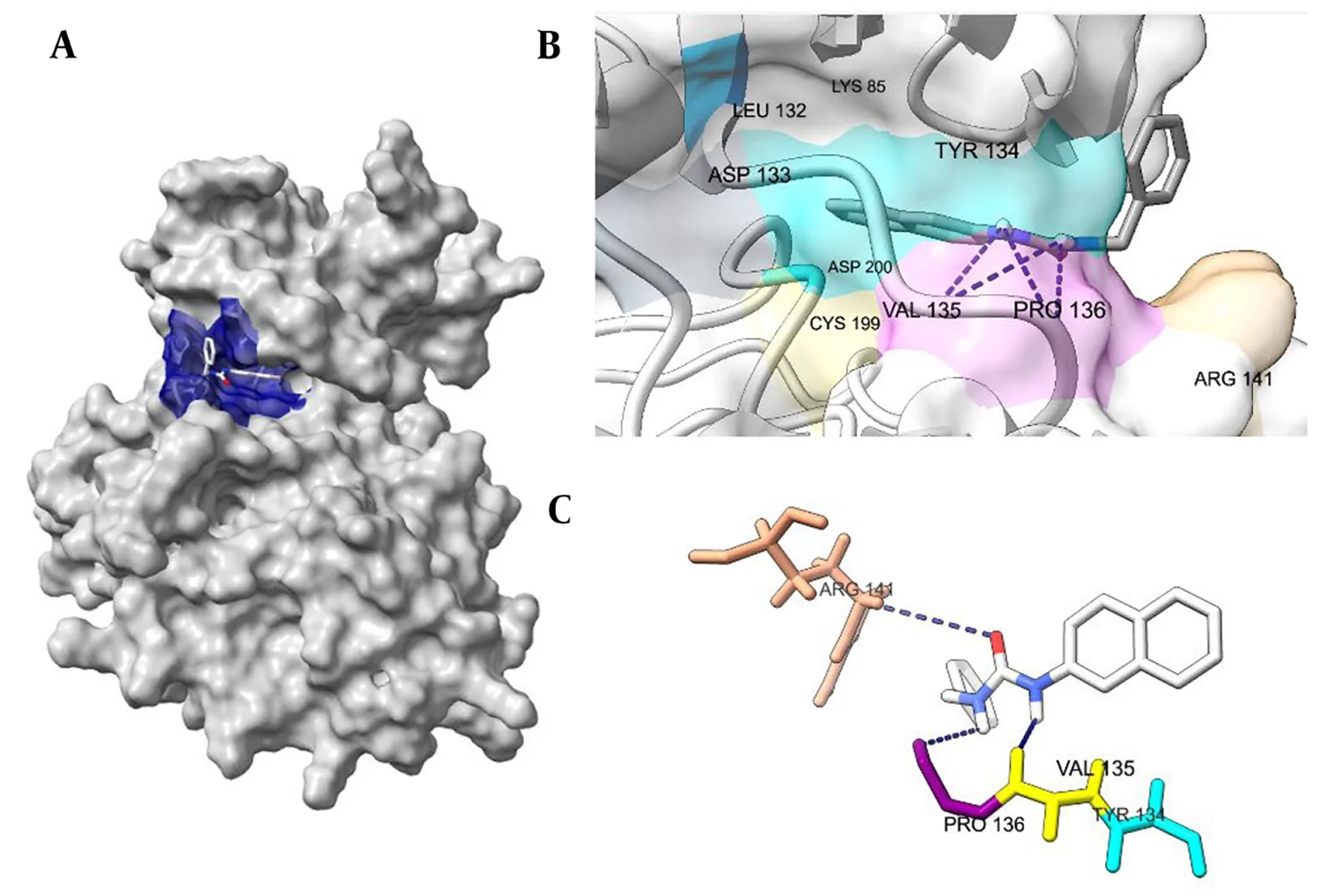
Binding interactions of compound 3b with the ATP-binding site of GSK-3β (PDB ID: 1Q5K). A, Surface view showing compound 3b within the ATP-binding cavity. B and C, Close-up views of the active site showing compound 3b and key interactions with Pro136, Val135, and Arg141.

The hydrogen-bond donor and acceptor groups of the urea core in all 17 derivatives formed hydrogen bonds with Tyr134, Val135, or Pro136 (39). This interaction pattern, shown in Figure 4, is characteristic of ATP-competitive GSK-3β inhibitors and allows the ligands to mimic ATP binding, thereby increasing binding affinity.

Docking results also indicated that Lys85 was a key residue in ligand stabilization. In derivatives with R_1_ substitutions, particularly those with extended linkers at the para position, ligand orientation allowed polar or aromatic groups to approach Lys85, resulting in electrostatic or π-cation interactions. Arg141 and Tyr134 also contributed to kinase selectivity in several compounds, including dichloro and meta-chloro derivatives, through π-alkyl interactions. These auxiliary contacts, together with conserved hinge interactions, support further optimization of this scaffold for potent GSK-3β inhibition. All compounds showed substantial interactions with the GSK-3β hydrophobic pocket formed by Ile62, Val70, Ala83, Leu132, Leu188, and Cys199 (40, 41) (Figure 4).

### 4.3. Molecular Dynamics Analysis

**Figure 5.**
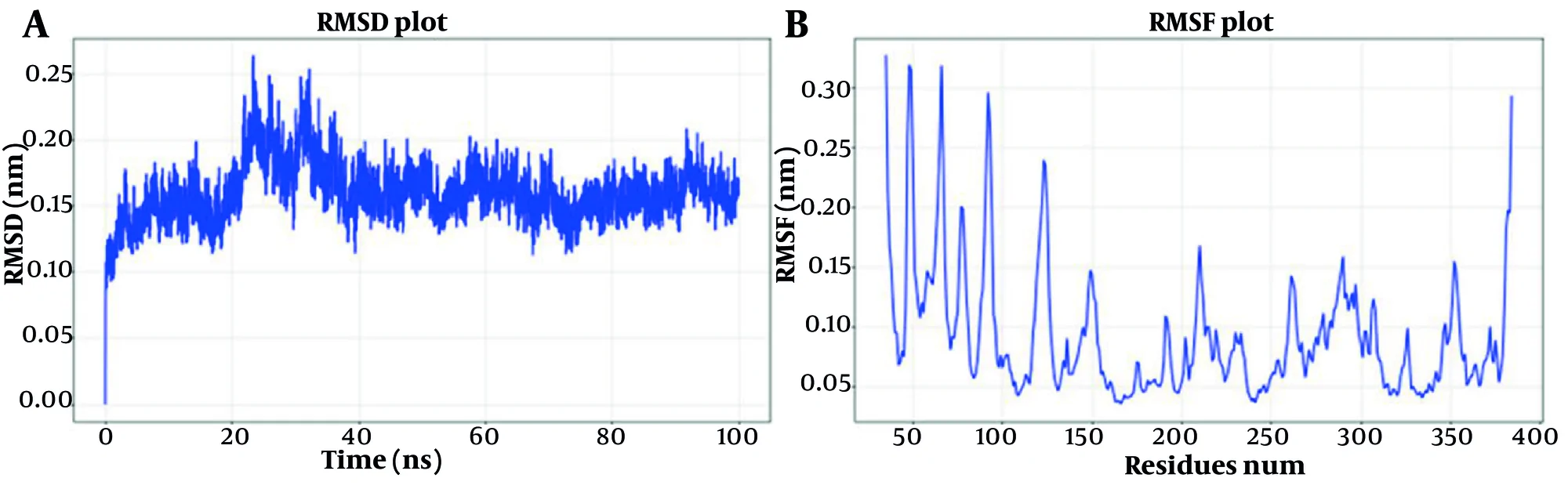
A, Root mean square deviation plot of the protein-ligand complex. B, Root mean square fluctuation of residues in the protein structure.

The RMSD profile showed a prominent structural shift between 20 and 40 ns, suggesting a conformational rearrangement within the complex. After this transition, the system stabilized at 0.142 ± 0.029 nm and maintained a steady RMSD trajectory through 100 ns, indicating regained structural integrity (42). RMSF analysis showed that residues 35 - 150 exhibited the greatest flexibility, consistent with their location in loop regions. In contrast, active-site residues Lys60, Val61, Ile62, Val70, Gln72, Ala83, Lys85, Val110, Leu132, Asp133, Tyr134, Pro113, Glu113, Arg141, Gln185, Leu188, Cys199, and Asp220 had low RMSF values (< 0.3 nm), indicating stabilization after ligand binding (43, 44). Binding affinity was calculated using MM-GBSA with frames extracted from the stabilized phase. Trajectories from 40 to 100 ns were sampled at 1-ns intervals for free-energy calculations. The MM-GBSA binding free energy (ΔG) was -55.30 ± 1.3 kcal/mol, indicating strong affinity and the formation of a stable protein-ligand complex. Detailed data are provided in Figures S4-S6 in the Supplementary File (Figure 5).

### 4.4. In Vivo Behavioral Evaluation in the STZ-Induced Alzheimer Disease Model

#### 4.4.1. Open-Field Test

**Figure 6.**
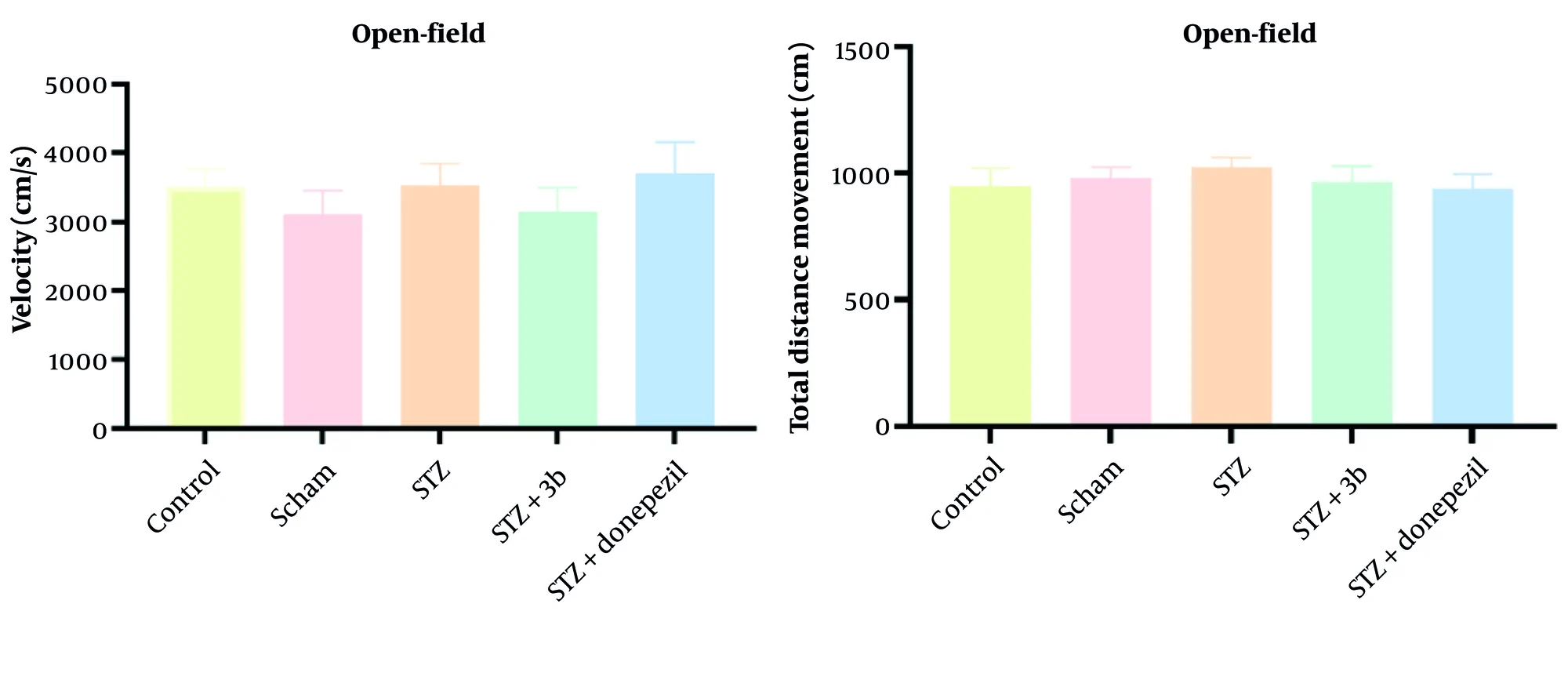
Effects of compound 3b (10 mg/kg) on A, velocity (cm/s), and B, total distance traveled (cm) in the open-field test. The Alzheimer disease model was induced by streptozotocin (STZ; 3 mg/kg). Data were collected over 28 consecutive days after stereotaxic surgery and analyzed by 1-way analysis of variance followed by the Tukey test. Data are presented as mean ± SEM (n = 8).

In the open-field test, administration of compound 3b to STZ-treated animals did not significantly change the total distance traveled or velocity compared with the control and sham groups (Figure 6). These findings indicate that treatment had no significant effect on locomotor activity at the dose used in this study.

#### 4.4.2. Radial Arm Water Maze Test

In the RAWM test, Figure S5A in the Supplementary File showed that the STZ group had a significant increase in latency to locate the platform compared with the sham group. The donepezil group had a shorter latency than the STZ group. Treatment with compound 3b also significantly reduced latency compared with the STZ group across trial days (F(36, 350) = 4.146; P < 0.0001). Figure S5B in the Supplementary File showed that the STZ group had significantly more reference-memory errors than the sham group. The donepezil group had fewer reference-memory errors than the STZ group. Treatment with compound 3b also significantly reduced the number of reference-memory errors compared with the STZ group across trial days (F(36, 350) = 1.531; P < 0.0001). Detailed animal-model data are provided in Figures S7 and S8 in the Supplementary File.

## 5. Discussion

### 5.1. Conclusions

In this study, a new series of 17 1-benzyl-3-phenylurea derivatives was designed and evaluated as potential GSK-3β inhibitors for the treatment of AD. Molecular docking analyses showed that these compounds interacted effectively with the ATP-binding pocket of GSK-3β and formed key hydrogen bonds in the hinge region, consistent with those of high-affinity ATP-competitive inhibitors. Among the synthesized derivatives, compound 3b exhibited the most promising profile, with favorable docking and molecular dynamics results and significant improvement in spatial learning and memory in the STZ-induced Alzheimer-like model. Overall, this chemical scaffold has molecular features suitable for further optimization toward potent, brain-penetrant GSK-3β inhibitors. Future work will focus on structural modifications to enhance selectivity, pharmacokinetic stability, and BBB permeability, as well as on validating the mechanism of action through enzymatic and molecular studies. These results provide a foundation for developing next-generation GSK-3β modulators with therapeutic potential in AD.

**Figure 7.**
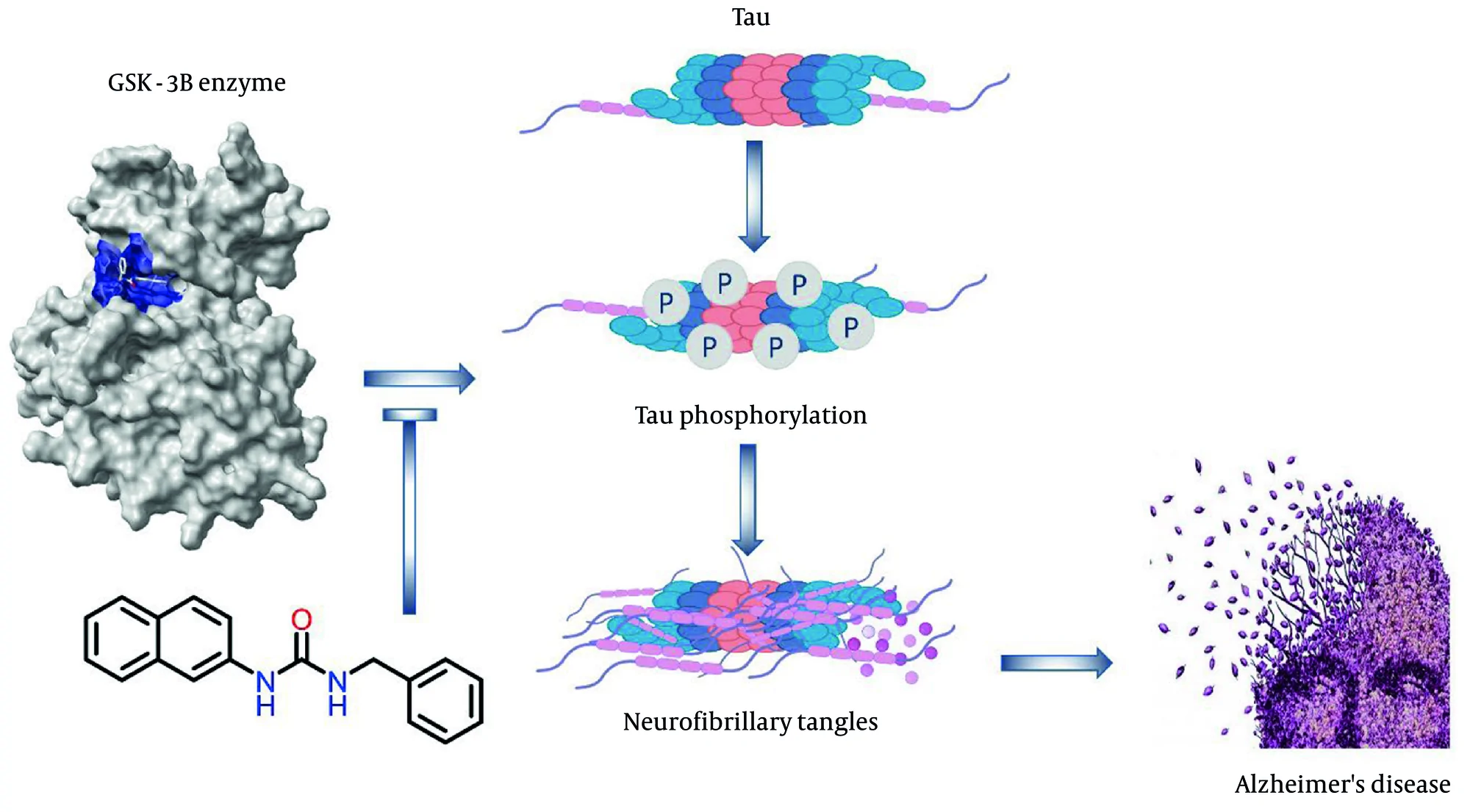
Schematic representation of GSK-3β-mediated tau phosphorylation leading to tau aggregation, neurofibrillary-tangle formation, and progression of Alzheimer disease. The illustrated small molecule targets GSK-3β as a potential inhibitor of this pathological pathway.

As illustrated in Figure 7, GSK-3β-mediated tau hyperphosphorylation promotes the formation of neurofibrillary tangles, a key pathological feature of AD.

ijpr-25-1-172672-s001.pdf

## Data Availability

The data presented in this study are provided in the main text and are openly available for readers upon request.
