# Supplementary material for: Design, Synthesis, Molecular Dynamics Analysis, and Biological Evaluation of New Urea Derivatives in an Animal Model of Alzheimer Disease
Source: Iran J Pharm Res. 2026 Aug 16;25(1):e172672. doi: 10.5812/ijpr-172672 (PMC13589810; doi:10.5812/ijpr-172672)
Supplement: Supplementary file 1 [file ijpr-25-1-172672-s001.pdf]

NMR Spectral Data

1-Benzyl-3-phenylurea (Compound 3a)

Yield: 95%; White powder; mp 240 °C; FT-IR (KBr, cm^{-1}): 3339, (N–H stretching), 1610 (C=O stretching); LC-MS (ESI): m/z 227.1 [$M+H^+$], 249 [$M+Na^+$]; ^{1}HNMR (400 MHz, DMSO-d_6) δ (ppm): ^{1}H NMR (400 MHz, DMSO) 4.31 (2H, d, *J*=6.4 Hz, CH_2), 6.63 (1H, t, *J*=6.0 Hz, NH-CH_2), 6.90 - 7.43, 10H-Ar; 6.90 (1H, t, *J*=8.1 Hz, H_4 benzyl), (9H, $H_{2,3,5,6}$ benzyl, $H_{2,3,4,5,6}$ phenyl), 7.42 (1H, s, *J*=8.5 Hz, NH); ^{13}CNMR (100 MHz, DMSO-d_6) δ (ppm): 43.18, 118.13, 121.55, 127.19, 127.55, 128.78, 129.13, 140.83, 140.93, 155.7; Anal. Calcd. for $C_{14}H_{14}N_2O$: C, 74.31; H, 6.24; N, 12.38. Found:

1-Benzyl-3-(naphthalen-2-yl) urea (Compound 3b)

Yield: 90%; Purple powder; mp 255 °C; FT-IR (KBr, cm^{-1}): 3313 (N–H stretching), 1628 (C=O stretching); LC-MS (ESI): m/z 299 [$M+Na^+$]; ^{1}H NMR (400 MHz, DMSO), 4.37 (2H, d, *J*=7.2 Hz, CH_2), 7.07 (1H, t, *J*=6.2 Hz, NH-CH_2), 7.25-7.58 H-Ar(9H, m, $H_{2,3,4,5,6}$ phenyl, $H_{5,6,7,9,10}$ benzyl), 7.90 (1H, t, *J*=4.6 Hz, H_8 benzyl), 8.03 (1H, d, *J*=8.8 Hz, H_5 benzyl), 8.11(1H, d, *J*=9.4 Hz, H_2 benzyl), 8.63 (1H, s, NH); ^{13}CNMR (100 MHz, DMSO-d_6) δ (ppm): 43.43, 116.9, 121.78, 122.59, 125.93, 126.24, 126.41, 127.3, 127.71, 128.86, 134.19, 135.56, 140.67, 156; Anal. Calcd. for $C_{18}H_{16}N_2O$: C, 78.24; H, 5.84; N, 10.14. Found:

1-(1,1′-Biphenyl-4-yl)-3-benzylurea (Compound 3c)

Yield: 92%; White powder; mp 260 °C; FT-IR (KBr, cm^{-1}): 3331 (N–H stretching), 1628 (C=O stretching); LC-MS (ESI): m/z 303 [$M+H^+$]; ^{1}H NMR (400 MHz, DMSO) 4.24 (2H, d, *J*=6.0 Hz, CH_2), (7.03 (1H, t, *J*=6.3 Hz, NH-CH_2), 7.05-7.90 14H-Ar; 7.08 (1H, t, *J*=7.8 Hz, H_4 benzyl), 7.16 (1H, dd, *J*=1.0, 7.8 Hz, H_2 benzyl), 7.25 (3H, d, *J*=7.4 Hz, $H_{2,3,5}$ biphenyl), 7.38-7.29 (5H, m, $H_{3,5,6}$ benzyl, $H_{8,9}$ biphenyl),7.48 (3H, t, *J*=8.0 Hz, $H_{9,10,11}$ biphenyl), 7.90 (1H, d, *J*=9.0 Hz, H_{12} biphenyl). Anal. Calcd. for $C_{20}H_{18}N_2O$: C, 79.44; H, 6.00; N, 9.26. Found:

1-Benzyl-3-(p-tolyl) urea (Compound 3d)

Yield: 99%; white powder; mp 200 °C; FT-IR (KBr, cm^{-1}): 3309 (N–H stretching), 1632 (C=O stretching); LC-MS (ESI+): m/z 241 [$M+H^+$]; ^{1}H NMR (400 MHz, DMSO) 2.21 (3H, s, CH_3), 4.29 (2H, d, *J*=5.7 Hz, CH_2), 6.55 (1H, t, *J*=5.0 Hz, NH-CH_2), 7.02-7.04, 9H-Ar; (2H, d, *J*=9.0 Hz, $H_{2,6}$ benzyl),(7H,m, $H_{3,4,5}$ benzyl, $H_{2,3,5,6}$ phenyl) 8.43 (1H, s, NH); ^{13}CNMR (100 MHz, DMSO-d_6) δ (ppm): 20.7, 43.19, 118.2, 127.0, 127.5, 128.7, 129.5, 130.2, 138.3, 140.9, 155.7; Anal. Calcd. for $C_{15}H_{16}N_2O$: C, 74.97; H, 6.71; N, 11.66. Found:

1-Benzyl-3-(4-methoxyphenyl) urea (Compound 3e)

Yield; 95%; White powder; mp 211 °C; FT-IR (KBr, cm⁻¹): 330 (N–H stretching), 1610 (C=O stretching); LC-MS (ESI): m/z 279 $[M+Na^+]$; ^{1}H NMR (400 MHz, DMSO) 3.69 (3H, s, OCH_3), 4.28 (2H, d, *J*=5.3 Hz, CH_2), 6.54 (1H, t, *J*=6.4 Hz, NH-CH_2), 6.80-7.35, 9H-Ar; 6.82 (2H, d, *J*=8.4 Hz, $H_{2,6}$ benzyl), 7.25 (1H, t, *J*=7.2 Hz, H_4 benzyl), 7.32 (6H, m, $H_{3,5}$ benzyl, H $_{2,3,5,6}$ H-phenyl), 8.39 (1H, s, NH); ^{13}CNMR (100 MHz, DMSO-d_6) δ (ppm): 55.58, 43.2, 114.3, 119.8, 127.1, 127.5, 128.7, 134, 141, 154.3, 155.9; Anal. Calcd. for $C_{15}H_{16}N_2O_2$: C, 70.29; H, 6.29; N, 10.93. Found:

1-Benzyl-3-(4-fluorophenyl) urea (Compound 3f)

Yield: 95%; Golden powder; mp 181–183°C; FT-IR (KBr, cm⁻¹): 3291(N–H stretching), 1625 (C=O stretching); LC-MS (ESI): m/z 245 $[M+H^+]$; ^{1}HNMR (400 MHz, DMSO-d_6) δ (ppm): 4.29 (d, 2H, *J* = 5.1 Hz, CH_2), 6.96 (t, *J* = 8 Hz, 1H, NH-CH_2), 7.11-7.45, H-Ar; 7.12 (t, 2H, *J* = 8.4Hz, $H_{3.4}$ benzyl), 7.28 (t, 2H, *J* = 8.4 Hz, H_5 benzyl, H_3 phenyl), 7.45 (m, 4H, $H_{2,3,5,6}$ phenyl), 8.66 (1H,s, NH); ^{13}CNMR (100 MHz, DMSO-d_6) δ (ppm): 43.19, 116.6, 127.1, 127.2, 129.3, 129.4, 129.5, 129.7, 130.9, 152.9; Anal. Calcd. for $C_{14}H_{13}FN_2O$: C, 68.84; H, 5.36; N, 11.47. Found:

1-Benzyl-3-(4-chlorophenyl) urea (Compound 3g)

Yield: 97%; White powder; mp 190 °C; FT-IR (KBr, cm⁻¹): 3313 (N–H stretching), 1632 (C=O stretching); LC-MS (ESI): m/z 259 $[M-H]^-$; ^{1}HNMR (400 MHz, DMSO-d_6) δ (ppm): 4.29 (2H, d, *J*=5.9 Hz, CH_2), 6.67 (1H, t, *J*=6.1 Hz, NH-CH_2), 7.22-7.46; 9H-Ar; (7H, m, $H_{2,3,4,5,6}$ benzyl, $H_{3,5}$ phenyl, 7.44 (2H, d, *J*=9.6 Hz, H $_{2,6}$ phenyl), 8.72 (1H, s, NH); ^{13}CNMR (100 MHz, DMSO-d_6) δ (ppm): 43.2, 119.6, 124.9, 127.2, 127.5, 128.7, 128.9, 139.9, 140.6, 155.5; Anal. Calcd. for $C_{14}H_{13}ClN_2O$: C, 64.50; H, 5.03; N, 10.74. Found:

1-Benzyl-3-(4-bromophenyl) urea (Compound 3h)

Yield: 96%; White powder; mp 196–197 °C; FT-IR (KBr, cm⁻¹): 3313 (N–H stretching), 1632 (C=O stretching); LC-MS (ESI): m/z 306.9 $[M+H^+]$; ^{1}HNMR (400 MHz, DMSO-d_6) δ (ppm): 4.29 (2H, d, *J*=5.9 Hz, CH_2), 6.68 (1H, t, *J*=6.6 Hz, NH-CH_2), 7.24-7.39, H-Ar;
7.24 (1H, t, *J*=7.4 Hz, H_4 benzyl),7.29 - 7.39 (9H, m, H $_{2,3,4,5,6}$ benzyl, $H_{2,3,5,6}$ phenyl); 8.74 (1H, s, NH);^{13}CNMR (100 MHz, DMSO-d_6) δ (ppm): 43.1, 112.8, 120, 127.2, 127.5, 128.7, 131.8, 140.3, 140.6, 155.5; Anal. Calcd. for $C_{14}H_{13}BrN_2O$: C, 55.10; H, 4.29; N, 9.18. Found:

1-Benzyl-3-(4-(methylthio) phenyl) urea (Compound 3i)

Yield: 95%; White powder; mp 166–167 °C; FT-IR (KBr, cm⁻¹): 3317 (N–H stretching), 1632 (C=O stretching); LC-MS (ESI): m/z 271 $[M-H]^-$; ^{1}HNMR (400 MHz, DMSO-d_6) δ (ppm): 2.41(3H, s, SCH_3), 4.24 (2H, d, *J*=5.8 Hz, CH_2), 6.47 (1H, t, *J*=6.4 Hz, NH-CH_2), 7.18 (9H, m, $H_{2,3,5,6}$

benzyl, $H_{2,3,5,6}$ phenyl) 8.65 (1H, s, NH);^{13}CNMR (100 MHz, DMSO-d_6) δ (ppm): 16.65, 43.2, 118.89, 127.2, 127.5, 127.7, 128, 128.9, 138.9, 140.6, 155.5; Anal. Calcd. for $C_{15}H_{16}N_2OS$: C, 66.15; H, 5.92; N, 10.29. Found:

1-Benzyl-3-(o-tolyl) urea (Compound 3j)

Yield: 93%; White powder; mp 160 °C; FT-IR (KBr, cm^{-1}): 3317 (N–H stretching), 1628 (C=O stretching); LC-MS (ESI): m/z 241 [M+H$^+$]; ^{1}HNMR (DMSO-d_6, 400 MHz) δ (ppm): 2.18 (s, 3H, CH_3), 4.30 (2H, d, J=5.2 Hz, CH_2), 6.88 (1H, t, J=6.8 Hz, NH-CH_2), 6.99-7.85 (H-Ar), 7.00 (1H, t, J=5.9 Hz, H_4 benzyl), 7.11 (2H, dd, J=7.9, 15.3 Hz, $H_{4,6}$ benzyl), 7.24-7.36 (5H, m, H $_{3,5}$ benzyl, H 4,5,6 phenyl), 7.72 (1H, s, H_3 phenyl), 7.83 (1H, s, NH);^{13}CNMR (100 MHz, DMSO-d_6) δ (ppm): 18.3, 43.3, 120.8, 122.3, 126.5, 127.2, 127.7, 128.8, 130.5, 138.6, 140.7, 155.8; Anal. Calcd. for $C_{15}H_{16}N_2O$: C, 74.97; H, 6.71; N, 11.66. Found:

1-Benzyl-3-(3-methoxyphenyl) urea (Compound 3k)

Yield: 94%; White powder; mp 190 °C; FT-IR (KBr, cm^{-1}): 3321 (N–H stretching), 1643 (C=O stretching); LC-MS (ESI): m/z 279 [M+Na$^+$]; ^{1}HNMR (400 MHz, DMSO-d_6) δ (ppm): 3.70 (3H, s, CH_3), 4.30 (2H, d, J=5.3 Hz, CH_2), 6.63 (1H, t, J=6.3 Hz, NH-CH_2), 6.88-7.36, H-Ar; 6.8 (1H, d, J=8.1 Hz, H_6 benzyl),7.13 (1H, dd, J=9.6, 17.0 Hz, H_2 benzyl), 7.36 - 7.21 (7H, m, $H_{3,4,5}$benzyl, H $_{2,4,5,6}$ phenyl), 8.59 (1H, s, NH);^{13}CNMR (100 MHz, DMSO-d_6) δ (ppm): 43.1, 55.2, 103.8, 107, 110.4, 127.5, 128.7, 140.8, 155.6, 160.1; Anal. Calcd. for $C_{15}H_{16}N_2O_2$: C, 70.29; H, 6.29; N, 10.93. Found:

1-Benzyl-3-(3-chlorophenyl) urea (Compound 3l)

Yield: 96%; White powder; mp 181–183 °C; FT-IR (KBr, cm^{-1}): 3317 (N–H stretching), 1632 (C=O stretching); LC-MS (ESI): m/z 259 [M-H]$^-$; ^{1}HNMR (400 MHz, DMSO-d_6) δ (ppm): 4.30 (2H, d, J=7.9 Hz, CH_2), 6.72 (1H, t, J=6.2 Hz, NH-CH_2), 6.94-7.69, H-Ar; (1H, d, J=6.2 Hz, H $_{2,6}$ benzyl), 7.28-7.17 (3H, m, $H_{3,4,5}$ benzyl), 7.36 - 7.28 (4H, m, $H_{3,4,5,6}$ phenyl), 7.69 (1H, s, H_2 phenyl), 8.81 (1H, s, NH);^{13}CNMR (100 MHz, DMSO-d_6) δ (ppm): 43.1, 116.5, 117.4, 121.1, 127.5, 128.7, 130.7, 133.5, 140.6, 142.4, 155.4; Anal. Calcd. for $C_{14}H_{13}ClN_2O$: C, 64.50; H, 5.03; N, 10.74. Found:

1-Benzyl-3-(3,4-dimethylphenyl) urea (Compound 3m)

Yield: 95%; White powder; mp 141 °C; FT-IR (KBr, cm^{-1}):3302 (N–H stretching), 1622 (C=O stretching); LC-MS (ESI): m/z 255 [M+H$^+$]; ^{1}H NMR (400 MHz, DMSO) 2.08 (3H, s, CH_3), 2.24 (3H, s, CH_3), 4.29 (2H, d, J=9.4 Hz, CH_2), 6.88 (2H, t, J=6.1 Hz, NH-CH_2), 6.83-7.53 H-Ar; (2H, d,t, J=7.2, J=9.5 Hz, NH-CH_2, H_2 benzyl), 6.98 (1H, t, J=8.1 Hz, H_4 benzyl), 7.25 (1H, t, J=7.3 Hz, H_3 benzyl), 7.34 (4H, m, 10.8 Hz, $H_{5,6}$ benzyl, $H_{5,4}$ phenyl), 7.53 (1H, d, J=5.9 Hz, H_2 phenyl), 7.74 (1H, s, NH); ^{13}CNMR (100 MHz, DMSO-d_6) δ (ppm): 14.1, 20.8, 43.3, 120.3,

124.7, 124.6, 125.6, 127.2, 127.6, 128.8, 136.8, 138.2, 140.8, 156.1; Anal. Calcd. for $C_{16}H_{18}N_2O$: C, 75.56; H, 7.13; N, 11.01. Found:

1-Benzyl-3-(2,6-dimethylphenyl) urea (Compound 3n)

Yield: 93%; White powder; mp 141 °C; FT-IR (KBr, cm^{-1}): 3309 (N–H stretching), 1632 (C=O stretching); LC-MS (ESI): m/z 255 $[M+H^+]$; ^1H NMR (400 MHz, DMSO) 2.18 (6H, s, $2CH_3$), 4.28 (2H, d, *J*=7.1 Hz, CH_2), (1H, t, *J*=13.8 Hz, NH-CH_2), 6.55-7.35 H-Ar; (3H, m, $H_{2,3,4}$ benzyl), 7.24 (5H, m, $H_{5,6}$ benzyl, $H_{3,4,5}$ phenyl),7.56 (1H, s, NH);^{13}CNMR (100 MHz, DMSO-d_6) δ (ppm): 18.7, 43.4, 126.1, 127, 127.3, 128.1, 128.6, 136.1, 136.4, 141.4, 156.5; Anal. Calcd. for $C_{16}H_{18}N_2O$: C, 75.56; H, 7.13; N, 11.01. Found:

1-benzyl-3-(3,4-dimethoxyphenyl) urea (Compound 3o)

Yield: 90%; Purple powder; mp 160 °C; FT-IR (KBr, cm^{-1}): 3328 (N–H stretching), 1632 (C=O stretching); LC-MS (ESI): m/z 287 $[M+H^+]$; ^1H NMR (400 MHz, DMSO) 3.72 (6H, s, $2OCH_3$), 4.30 (2H, d, *J*=5.8 Hz, CH_2), 6.96 (1H, t, *J*=6.6 Hz, NH-CH_2), 6.87-7.45 H-Ar; 6.87 (2H, d, *J*=2.0 Hz, $H_{2,6}$ benzyl), 7.20 (1H, s, H_2 phenyl), 7.26 (2H, t, *J*=9.4 Hz, $H_{3,5}$ benzyl), 7.45 (2H, d, *J*=7.4 Hz, $H_{2,3}$phenyl), 8.50 (1H, s, benzyl), 8.57 (1H, s, NH);^{13}CNMR (100 MHz, DMSO-d_6) δ (ppm): 43.2, 55.5, 62.5, 118.2, 119.1, 122.7, 127.2, 127.6, 128.7, 130.8, 131.3, 140.1, 140.5, 165.3; Anal. Calcd. for $C_{16}H_{18}N_2O_3$: C, 67.12; H, 6.34; N, 9.78. Found:

1-Benzyl-3-(3,4-dichlorophenyl) urea (Compound 3p)

Yield: 90%; White powder; mp 200 °C; FT-IR (KBr, cm^{-1}): 3328 (N–H stretching), 1632 (C=O stretching); LC-MS (ESI): m/z 296 $[M+H^+]$; ^1H NMR (400 MHz, DMSO) 4.30 (2H, d, *J*=5.8 Hz, CH_2), 6.81 (1H, t, J=5.9 Hz, NH-CH_2), 7.36 - 7.87, H-Ar; (6H, m, $H_{3,4,5,6}$ benzyl, $H_{3,4}$ phenyl), 7.46 (1H, d, *J*=9.2 Hz, H_2 benzyl), 7.87 (1H, d, *J*=2.4 Hz, H_2 phenyl), 8.93 (1H, s, NH);^{13}CNMR (100 MHz, DMSO-d_6) δ (ppm): 43.2, 118.2, 119.1, 122.7, 127.6, 128.7, 130.8, 131.3, 140.1, 140.5, 165.3; Anal. Calcd. for $C_{14}H_{12}Cl_2N_2O$: C, 56.97; H, 4.10; N, 9.49. Found:

1-benzyl-3-(3,4,5-trimethoxyphenyl) urea (Compound 3q)

Yield: 90%; White powder; mp 170 °C; FT-IR (KBr, cm^{-1}): 3294(N–H stretching), 1602 (C=O stretching); LC-MS (ESI): m/z 287 $[M+H^+]$; ^1H NMR (400 MHz, DMSO) 3.58 (3H, s, OCH_3), 3.72 (6H, s, CH_3), 4.30 (2H, d, *J*=7.1 Hz, CH_2), 6.58 (1H, t, *J*=6.2 Hz, NH-CH_2), 6.77-7.35, H-Ar; 6.76 (2H, s, $H_{2,6}$ phenyl), 7.25 (1H, t, *J*=7.0 Hz, H_4 benzyl), 7.36 - 7.27 (4H, m, $H_{2,3,5,6}$), 8.55 (1H, s, NH);^{13}CNMR (100 MHz, DMSO-d_6) δ (ppm): 43.2, 59, 60.5, 95, 127.1, 127.4, 128.7, 132.2, 137.1, 140.9, 153.2, 155.6; Anal. Calcd. for $C_{17}H_{20}N_2O_4$: C, 64.54; H, 6.37; N, 8.86.

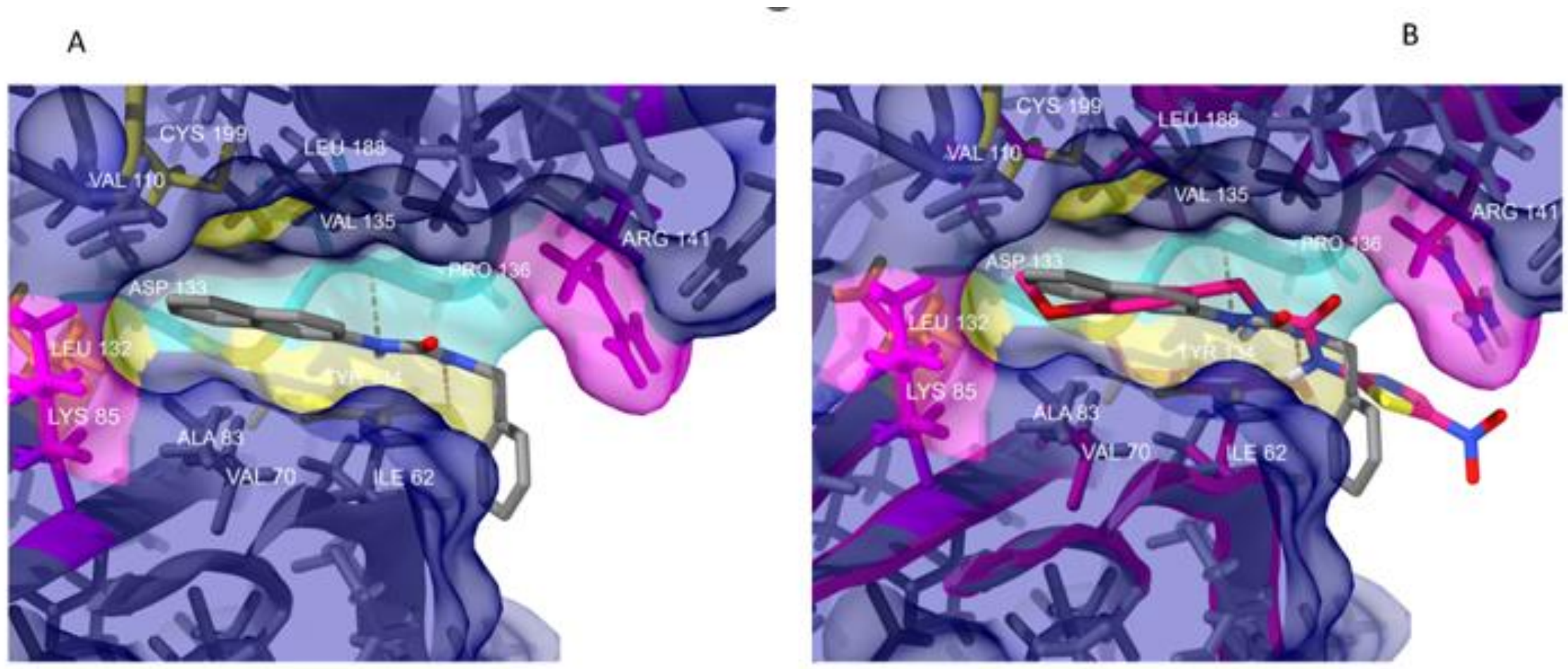

Figure S1. Structure (A).3b compound in the active site of GSK-3β (B). Overlap of the co-crystal structure of TMU and 3b compound in the ATP binding site of GSK-3β (C). Pink and yellow arrows show the unique residue of GSK-3β, and cyan arrows show the active site residue critical for inhibitory activity. UCSF ChimeraX version 1.10.1

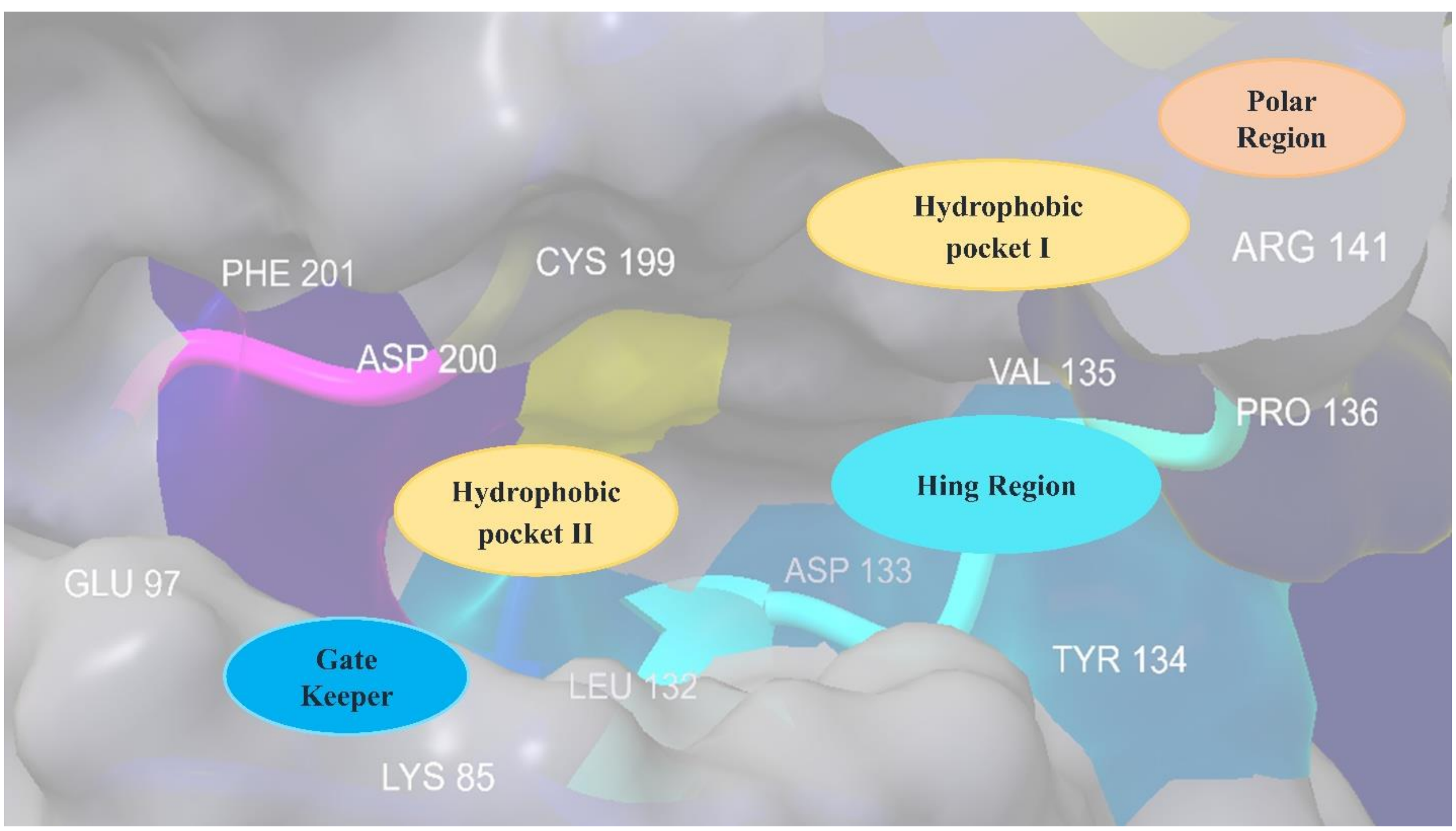

Figure S2.ATP binding pocket. The hinge region ensures molecular recognition, the hydrophobic back pocket I, II provides selectivity, the polar region enhances potency, and the solvent accessible region allows optimization of drug-like characteristics. This sub division of the ATP pocket forms the basis of modern structure-guided design of selective GSK-3β inhibitors.

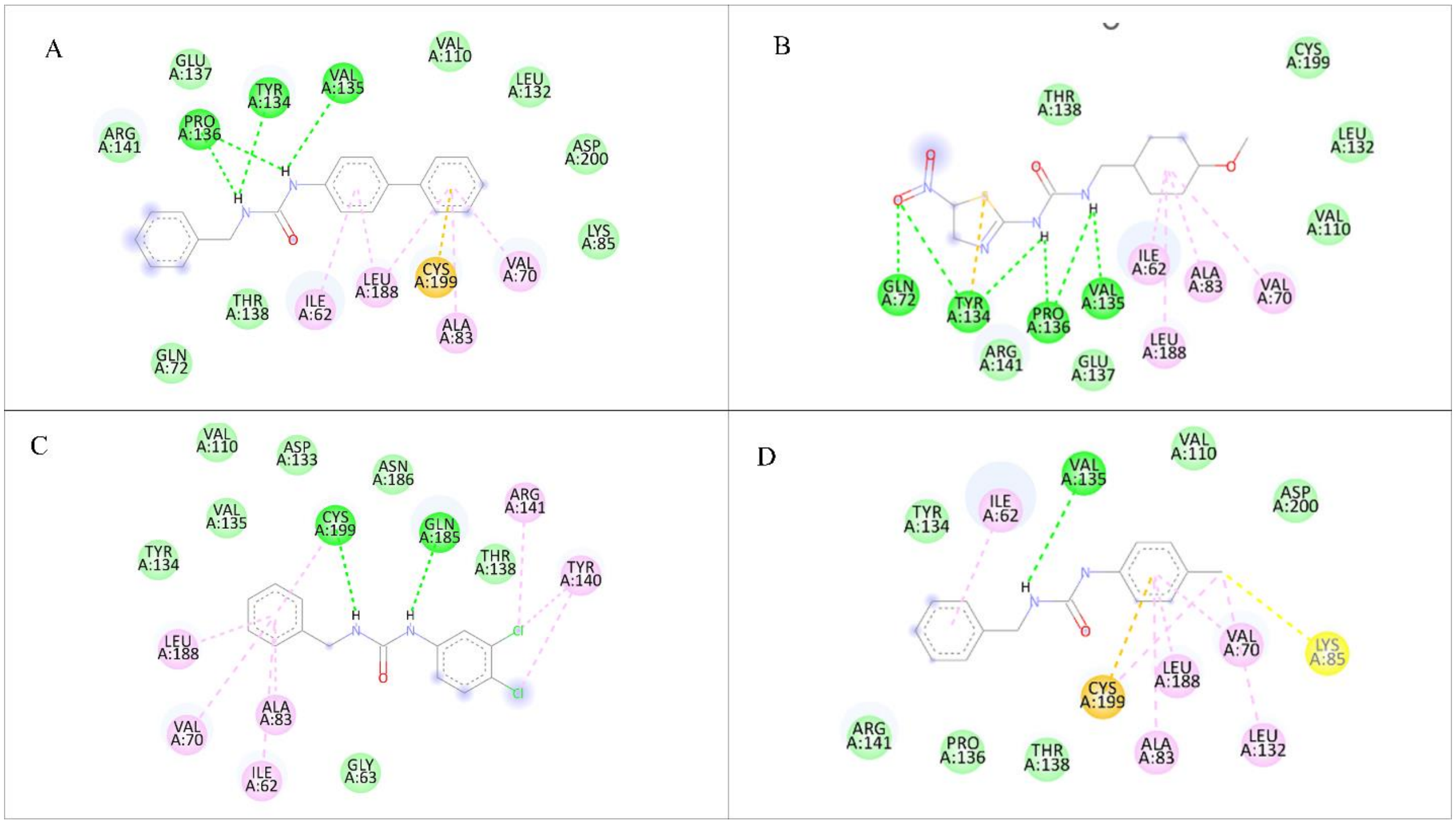

Figure S3. Docking poses of compounds: A)3c, B) AR-1014418 in GSK-3β (PDB ID: 1QK5), C).3p, D). 3d.

Molecular dynamics simulation

The RMSD profile in Figure 3 demonstrates clear convergence. Following an initial period of conformational adjustment, the RMSD values plateau after 40 ns of simulation time. Beyond this threshold, the trajectory exhibits only marginal oscillations with reduced amplitude, indicative of a well-equilibrated system. The absence of any significant drift or abrupt structural transitions suggests that the system has reached a thermodynamically stable state, and the residual fluctuations are consistent with typical thermal noise. This plateau region confirms that the simulation has adequately sampled the equilibrium ensemble, providing a reliable basis for this research.

The SPC (Simple Point Charge) water model was selected due to its well-established performance in representing the structural and dynamic properties of proteins within the force field used, providing a good balance between computational efficiency and accuracy. Regarding

system convergence, as shown in the RMSD profile, the system exhibits clear convergence. Following an initial equilibration phase, the RMSD values plateau after approximately 40 ns. The absence of significant drift or abrupt transitions beyond this point indicates that the system has reached a stable equilibrium state, ensuring that the 100 ns production run is sufficient for reliable analysis.

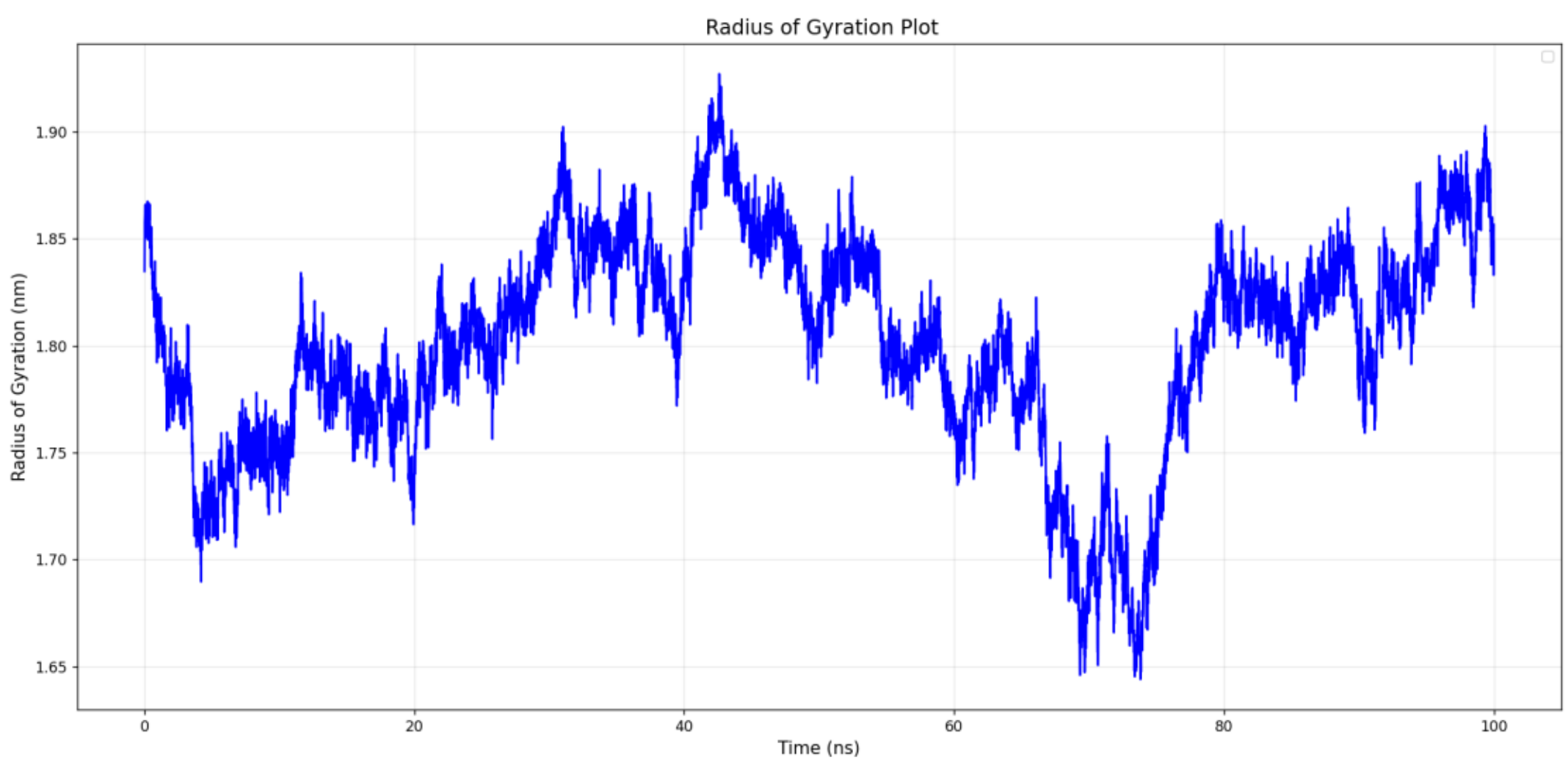

Figure S4. Radius of gyration.

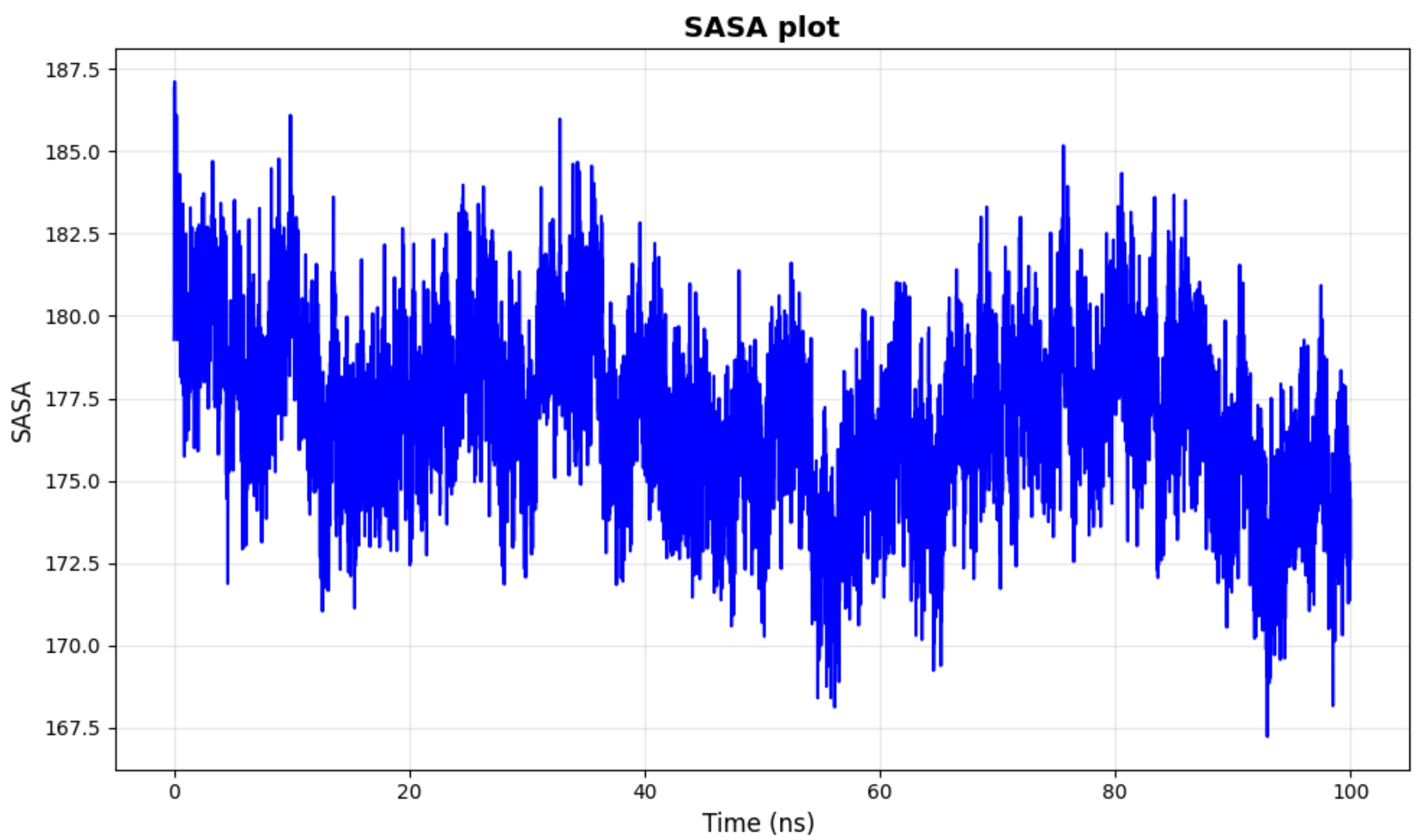

Figure S5. ligand-protein contact frequency.

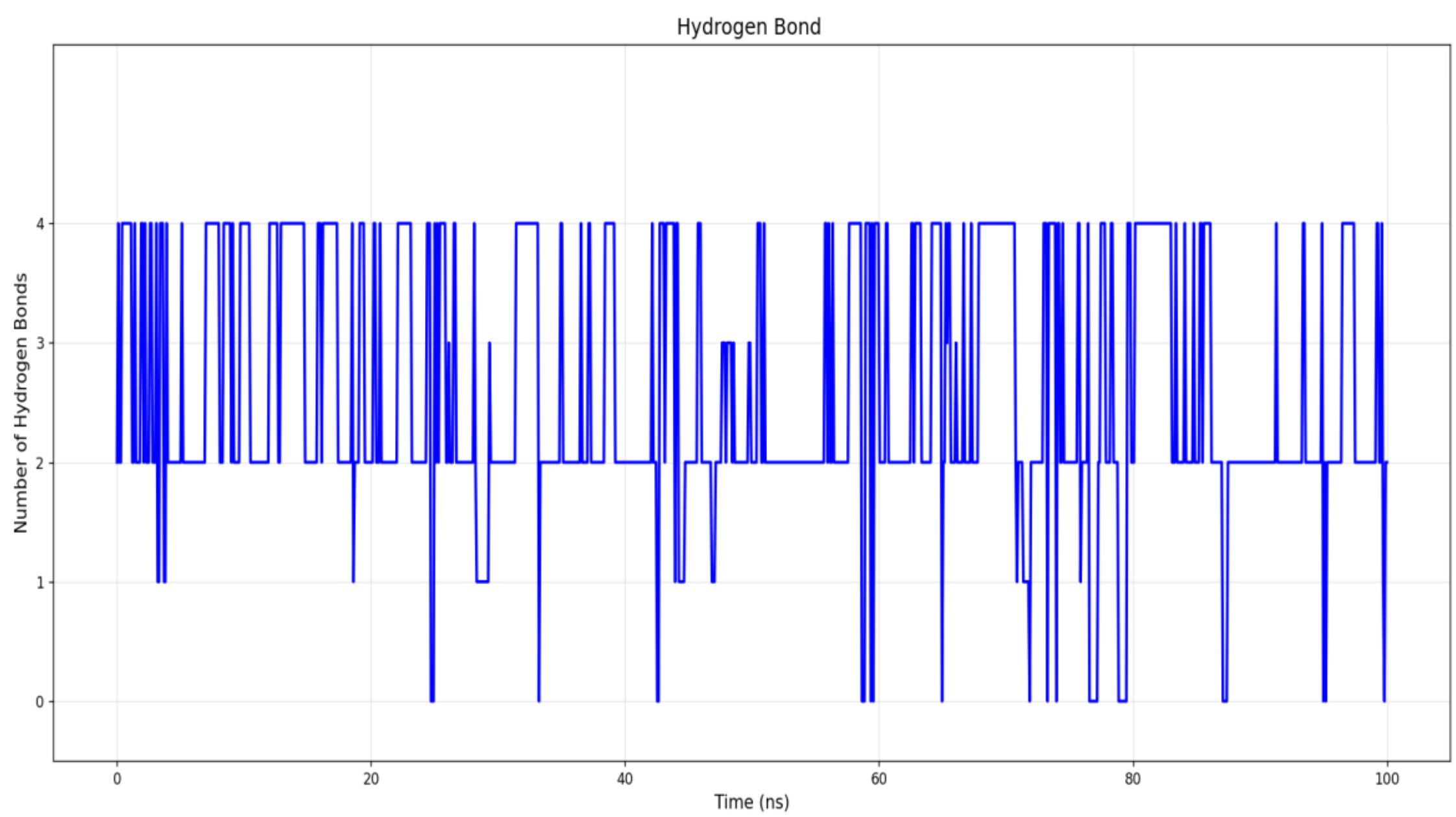

Figure S6. Hydrogen bond occupancy analyses.

In vivo evaluation

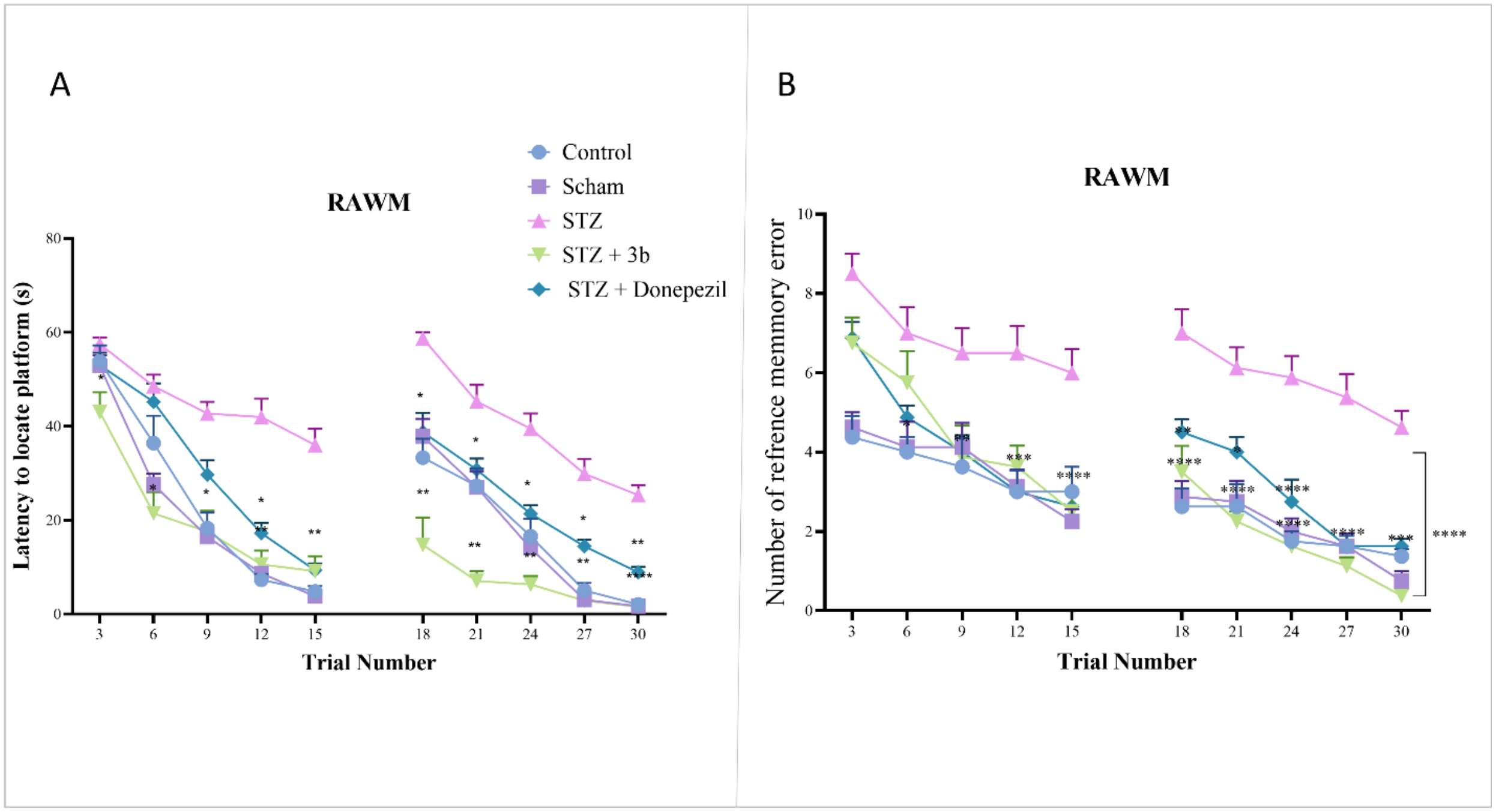

Figure S7. A. Effects of compound **3b** (10 mg/kg) on latency to locate the platform in the Radial Arm Water Maze test. B. Effects of compound **3b** (10 mg/kg) on the number of reference memory errors in the Radial Arm Water Maze test. The animal model of Alzheimer submitted by streptozotocin (STZ 3 mg/kg). Data were collected over 28 consecutive days after stereotaxic surgery. The results were analyzed by two-way ANOVA followed by Bonferroni's test. Data are described as mean ± SEM (n = 8). *P < 0.05, **P < 0.01, ***P < 0.001, ****P < 0.0001 compared to the STZ group.

On the probe day of RAWM test, the latency to enter the target arm, the number of entries into the target arm, and the duration in the target arm were assessed. Figure S8A. It was shown that the duration in the target arm was significantly reduced in the STZ group compared to the sham group ($p<0.0001$). The donepezil group noticed a significant increase in duration in the target arm compared to the STZ group ($p<0.0001$). Furthermore, treatment with compound **3b** significantly increased the duration in the target arm compared to the STZ group ($p<0.001$). Figure S8.B shown that the number of entries to the target arm during was significantly reduced in the STZ group compared to the sham groups ($p<0.01$). The donepezil group showed a significant increase in the number of entries into the target arm compared to the STZ group ($p<0.01$). Additionally, the treatment with compound **3b** significantly enhanced the number of entries into the target arm

compared to the STZ group (p<0.05). Figure S8.C shown that the latency to locate the target arm was significantly increase in the STZ group compared to the sham groups (p<0.0001). Furthermore, the donepezil group demonstrated a significant reduction in the latency to locate the target arm compared to the STZ group (p<0.0001). In addition, treatment with compound **3b** significantly decreased the latency to locate the target arm compared to the STZ group (p<0.0001).

In the probe trial, animals receiving the compound **3b** displayed significantly lower latency to locate the target arm, substantially increase the number of entries into the target arm and spent significantly more time in the target arm compared with the STZ group, indicating enhanced retention of spatial memory. Overall, treatment with the test compound effectively ameliorated STZ-induced deficits in spatial learning and memory in the rat model of AD.

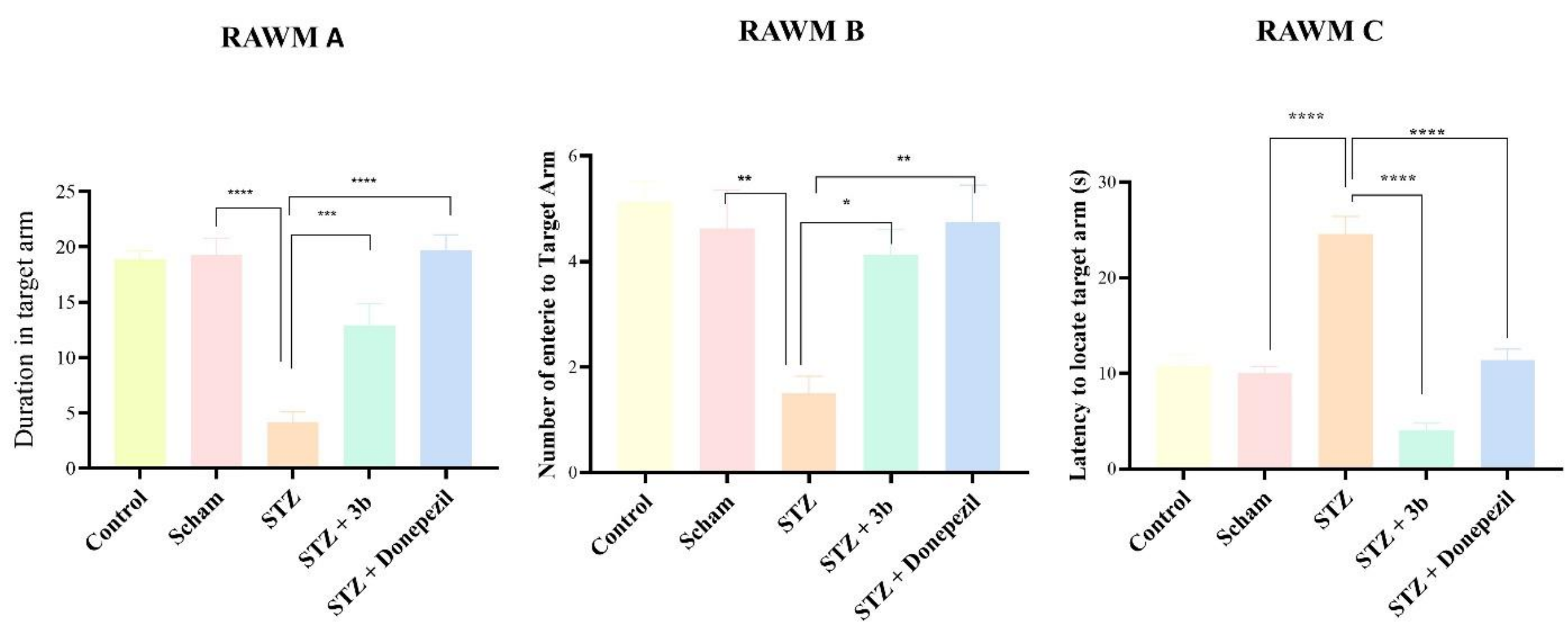

Figure S8. A. Effects of compounds **3b** (10 mg/kg) on duration in the target arm in the Radial Arm Water Maze test. B. Effects of compounds **3b** (10 mg/kg) on the number of entries to the target arm in the Radial Arm Water Maze test. C. Effects of compounds **3b** (10 mg/kg) on latency to locate the target arm in the Radial Arm Water Maze test. The animal model of AD was induced by streptozotocin (STZ 3 mg/kg). Data were collected over 28 consecutive days after stereotaxic surgery. The results were analyzed by one-way ANOVA followed by Tukey's test. Data are

described as mean ± SEM (n = 8). *P < 0.05, **P < 0.01, ***P < 0.001, ****P < 0.0001 compared to the STZ group.
